# Seasonality in diabetes in Yaounde, Cameroon: a relation with precipitation and temperature

**DOI:** 10.1186/s12889-016-3090-1

**Published:** 2016-06-06

**Authors:** Eric Lontchi-Yimagou, Maurice Tsalefac, Leonelle Monique Teuwa Tapinmene, Jean Jacques N. Noubiap, Eric Vounsia Balti, Jean-Louis Nguewa, Mesmin Dehayem, Eugene Sobngwi

**Affiliations:** Laboratory for Molecular Medicine and Metabolism, Biotechnology Center, University of Yaoundé I, Yaoundé, Cameroon; Departement of Geography, University of Yaoundé I, Yaoundé, Cameroon; Department of Medicine, Groote Schuur Hospital and University of Cape Town, Cape Town, South Africa; Medical Diagnostic Center, Yaoundé, Cameroon; Diabetes Research Center, Faculty of Medicine and Pharmacy, Brussels Free University, Brussels, Belgium; Department of Diabetes and Endocrinology, Hôpital Lariboisière, Assistance Publique-Hôpitaux de Paris, University Paris-Diderot Paris-7, Paris, France; National Obesity Center, Yaoundé Central Hospital, Yaoundé, Cameroon; Department of Internal Medicine and Specialties, Faculty of Medicine and Biomedical Sciences, University of Yaoundé I, Yaoundé, Cameroon

**Keywords:** Seasons, Diabetes, Yaounde, Cameroon, Sub-Saharan Africa

## Abstract

**Background:**

Diabetes is a growing health concern in developing countries, with Cameroon population having an estimated 6% affected. Of note, hospital attendees appear to be increasing all over the country, with fluctuating numbers throughout the annual calendar. The aim of the study was to investigate the relationship between diabete hospitalization admission rates and climate variations in Yaounde.

**Methods:**

A retrospectively designed study was conducted in four health facilities of Yaounde (Central Hospital, University teaching hospital, Biyem-Assi and Djoungolo District Hospitals), using medical records from 2000 to 2008. A relationship between diabetes (newly diagnosed diabetes patients or decompensated diabetics) hospitalization admissions and climate variations was determined using the “2000–2008” national meteorological database (precipitation and temperature).

**Results:**

The monthly medians of precipitation and temperature were 154mm and 25 °C, respectively. The month of October received 239mm of precipitation. The monthly medians of diabetic admissions rates (newly diagnosed or decompensated diabetes patients) were 262 and 72 respectively. October received 366 newly diagnosed diabetics and 99 decompensated diabetics. Interestingly, diabetic hospitalization admissions rates were higher during the rainy (51 %, 1633/3232) than the dry season, though the difference was non-significant. The wettest month (October) reported the highest cases (10 %, 336/3232) corresponding to the month with the highest precipitation level (239mm). Diabetes hospitalization admissions rates varied across health facilities [from 6 % (189/3232) in 2000 to 15 % (474/3232) in 2008].

**Conclusion:**

Diabetes is an important epidemiological disease in the city of Yaounde. The variation in the prevalence of diabetes is almost superimposed to that of precipitation; and the prevalence seems increasing during raining seasons in Yaoundé.

## Background

Diabetes mellitus, and type 2 diabetes (T2DM) in particular, is becoming increasingly important worldwide, assuming epidemic proportions in many populations, especially in developing countries including those in sub-Saharan Africa (SSA) [1, 2]. There is evidence that human activities have an effect on the climate of the planet, and they also have multiple effects on human health [3]. Gherard, in 1988, showed the impact of climate on health, particularly with a seasonal increase in mortality and morbidity [4]. Diabetes is a disease that affects the body’s metabolism, and so far science assigns no direct vector to the agent. The role of climate and environment in the pathogenesis of diabetes is only mentioned indirectly. Seasonality in the presentation of diabetes, especially Type 1 diabetes, has been recognized for many years. Type 2 diabetes has, however, also been shown to be associated with a seasonal incidence [5], suggesting that both types of diabetes may share common environmental precipitating factors. Several studies have shown that in the northern hemisphere the incidence of juvenile diabetes diagnosed during the fall or winter season were relatively higher than in the summer or spring [6]. The first report of seasonal variation in the new cases of diabetes was presented by Adams in the 1920s [7]. Since then, there have been a number of studies demonstrating seasonality in the time of clinical presentation of T1DM, although a consistent and integrated picture on the actual seasonality of the disease has not been established [8–20]. This seasonality variation has been taken as and indirect argument in favour of the environemental factors, it has been suggested that there is also a relationship between factors such as infections and vitamin D levels, and the seasonality of diabetes [21–27]. A recent study of the data on seasonal variation in diabetes in 53 countries has suggested that seasonality in the diagnosis of T1DM is indeed a real phenomenon and that this seasonality pattern appears to be related to the geographical location, at least as far as the northern/southern hemisphere is concerned [28]. The suggestion has also been made that metabolic changes during winter months could account for the seasonality of presentation [29]. Seasonal variation in the presentation of diabetes has not only been observed in the Northern Hemisphere but also in the Southern Hemisphere such as Australia [30], South Africa [31] and Tanzania [32]. However, data on many regions of the world are still lacking. According to an observation made in the National Obesity Centre of the Yaounde Central Hospital, Yaounde, Cameroon, more patients with diabetes are received during rainy seasons in Yaounde, being in a tropical environment. Therefore, this study was carried out to analyze the relationship between diabetes hospital admission rates (newly diagnosed diabetic or decompensated) and climate variations.

## Methods

### Ethics statement

The study was granted approval by the Institutional Review Board of the National Obesity Centre of the Yaounde Central Hospital. As the study is based on retrospective data, informed consent was not needed.

### Study population and settings

A retrospective study was conducted in four health facilities in Yaoundé, Cameroon, using hospital admission registers of diabetes patients from 2000 to 2008 hospitalized in the general medicine services of the Yaounde Central Hospital, the University teaching Hospital of Yaounde, the Djoungolo District Hospital and the Biyem-Assi District Hospital, and monthly data of precipitation and temperature over the same period (2000–2008) from the Weather Station of Nkolbisson, IRAD Yaounde, Cameroon.

### Measurements

#### Patients

Diabetes hospital admission rates were recorded using hospital admission registers of patients in general medicine in our study sites. Up to 31 December 2008, 3232 ethnic Africans were registered. Registration particulars included date of presentation to hospital. Diabetes was diagnosed based on a fasting plasma glucose >1.26g/l at least two times [33]. 74.7 % of newly diagnosed diabetes patients presented with classical symptoms of diabetes. Of the 3232 patients, 818 (25.3 %) were considered to have insulin-requiring diabetes on the basis of insulin need for control of symptoms and hyperglycaemia(decompensated diabetes) and where coming only from de Yaoundé Central Hospital.

#### Climate data

Climate variations were performed using the “2000–2008” national meteorological database from the weather station Nkolbisson IRAD Yaounde, Cameroon. This is the monthly data of precipitation and temperature over a period of 9 years (2000–2008).

### Definitions

The subjects were divided into groups according to the month of attendance to the hospital: January, February, March, April, May, June, July, August, September, October, November and December. Subjects were also grouped according to year (from 2000 to 2008) and the season they were attended in the hospital as follows: November to February (long dry season); March to June (long rainy season); July and August (short dry season); September and October (short rainy season) [34].

### Calculations

#### Average rain fall

$$ \mathrm{Pm}\left(2000\hbox{-} 2008\right)={\displaystyle \sum Px/n} $$

Pm: = Average precipitation(mm)
*n*: = Number of years, months, seasons
*Px*: = Amount of precipitation in a year x, a month x, a season x

#### IDFM: index of deviation from the mean

$$ \Delta \mathrm{x} = \mathrm{P}\mathrm{x}\ \hbox{--}\ \mathrm{P}\mathrm{m}\ \left(2000\hbox{-} 2008\right) $$

$$ \mathrm{If}\Delta \mathrm{x} = 0\ \mathrm{years} / ~\mathrm{month} /~ \mathrm{season}\ \mathrm{constant} $$

$$ \Delta \mathrm{x} < 0\ \mathrm{years}/~ \mathrm{month}/~ \mathrm{seasondeficit} $$

$$ \Delta \mathrm{x} > 0\ \mathrm{years}/~ \mathrm{month}/~ \mathrm{season}\ \mathrm{wet} $$

#### Rainfall index

$$ RI = Px/ Pm\ \left(2000-2008\right) $$

A year is described as wet if the ratio is greater than 1 and dry if it is less than 1.

## Results

During a 9 years period from 2000 to 2008, 3232 African diabetes patients were registered at University teaching Hospital, Yaounde Central Hospital, Djoungolo district Hospital, Biyem-Assi district Hospital, Cameroon. Newly diagnosed diabetes patients represented 74.7 % (*n* = 2414) of the study population whereas 25.3 % (*n* = 818) of participants where decompensated diabetes coming only from the Yaounde Central Hospital. The monthly medians of precipitation and temperature were estimate (min-max): 154 (9–239)mm and estimate (min-max): 25(24–26)°C, respectively (Table 1).

**Table 1:** General charactristics of the study

Variables	Values
Number of patients (n):	3232
Diabetes decompensation (n)	818
Overall average temperature rate °C (median; min; max)	(25; 24; 26)
Overall average precipitation rate mm (median; min; max)	(154; 9; 239)
Average seasonal precipitation rate mm (median; min; max)	
“Long dry season”	(25; 9; 99)
“Short rainy season”	(238; 237; 239)
“Long rainy season”	(186; 123; 199)
“Short dry season”	(154; 138; 169)

The number of diabetic patients registered to the Yaounde Central Hospital is 8 times higher than that recorded in the three combined study site. The Yaounde Central Hospital alone holds 88 % of diabetic patients in the city according to the results obtained in the study sites (Table 2).

**Table 2:** Number of registered diabetic patients per study location

Study Location	Number of Diabetics Registered	Overall average precipitation rate mm (median; min; max)
Central Hospital	2832	(154; 9; 239)
University teaching hospital	186	(154; 9; 239)
Biyem-Assi District Hospital	116	(154; 9; 239)
Djoungolo District Hospital	98	(154; 9; 239)

### Relationship between monthly precipitation, temperature and newly diagnosed and decompensated diabetes

**Fig. 1:**
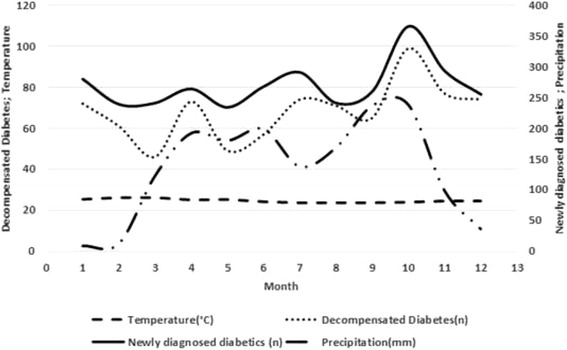
Monthly average number of patients with newly diagnosed and decompensated diabetes, and average temperature and precipitation rate decompensation diabetes, January 2000 to December 2008. Caption: 1 = January, 2 = February, 3 = March, 4 = April, 5 = May, 6 = June, 7 = July, 8 = August, 9 = September, 10 = October, 11 = November and 12 = December

The monthly medians of precipitation and temperature were estimate (min-max): 154 (9–239) mm and estimate (min-max): 24.7 (24–26)°C, respectively. The monthly medians (min-max) of hospital admission rates for newly diagnosed diabetes patients and decompensated diabetics were 262 (234–366) and 72 (46–99) people respectively. The highest level of precipitation was observed in October [estimate (min-max): 239 (9–239)mm] which coincided with the highest number of newly diagnosed [n (min-max): 366 (234–366)] and decompensated diabetes patients [n (min-max): 99 (46–99)] (Fig. 1).

### Relationship between seasonal precipitation, temperature and newly diagnosed and decompensated diabetes hospital admission rates

During the rainy seasons, the median of precipitation was estimate (min-max): 195.8 (123.2–238.6)mm compared to dry seasons estimate (min-max): 67.8 (9.1–168.9)mm. Seasonal data on diabetes hospitalization admissions rate at our study sites from 2002 to 2008 indicated that the greatest number of admissions occurred during the rainy seasons (51 %, 1633/3232) compared to the dry seasons (49 %, 1599/3232), though the difference was non-significant (Tables 3 and 4).

**Table 3:** Temperature, precititations and newly diagnosed and decompensated diabetes hospital admission rates during the four annual seasons

Seasons	Long dry season	Short dry season	Long rainy season	Shortrainy season
T (°C)	25 (25–26)	24 (24–24)	25 (24–26)	24 (24–24)
P (mm)	25 (9–99)	154 (138–169)	186 (123–199)	238 (237–239)
NDDP (n)	268 (239–293)	266 (241–291)	253 (234–268)	313 (260–366)
DD (n)	73 (61–77)	73 (71–74)	53 (46–73)	82 (65–99)

*NDDP* Newly Diagnosed Diabetic Patients, *DD* Decompensated Diabetes, November to February (long dry season); July and August (short dry season); March to June (long rainy season); September and October (shortrainy season)

Data are presented as median, minimun and maximun during the season

**Table 4:** Temperature, precititations and newly diagnosed and decompensated diabetes hospital admission rates during the four annual seasons

	Seasons		
	Dry season	Rainy season	*P*
T (°C) median (min-max)	25 (24–26)	25 (24–26)	0.879
P (mm) median (min-max)	68 (9–169)	196 (123–239)	0.005
NDDP (n)	1599	1633	0.802

Data are presented as median, minimun and maximun during the season

**Fig. 2:**
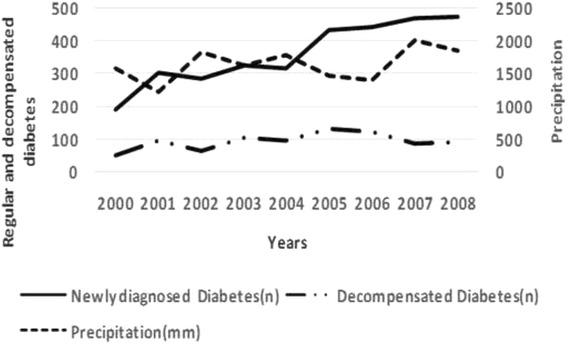
Relationship between annual precipitation, newly diagnosed and decompensated diabetes hospital admission rates

Diabetes hospitalization admissions rates varied across health facilities [from 6 % (189/3232) in 2000 to 15 % (474/3232) in 2008]. As depicted in Fig. 2, there was a constant increase of new cases of diabetes over time.

## Discussion

In this study, the number of hospitalizations related to newly diagnosed and decompensated diabetes evolved gradually between 2000 and 2008 in line with WHO estimates for developing countries (WHO, 2006). The major finding of this study is that, the variation in the incidence of diabetes is almost superimposed to that of precipitation, the hospital admission rate of diabetes patients is slightly higher during the rainy season. The month of October (the wettest month), reported the highest number of cases in the four health facilities involved in our study.

Knowing that glycemic control in diabetes depends not only on endogenous factors [35], but also has exogenous determinants [36], it is important to determine the nature of the evolution of newly and decompensated diabetes throughout months and seasons. This could allow to grasp the possible factors responsible for the seasonal incidence of the disease. The prevention of diabetes, including the environmental factors associated, may therefore need to be intensified and seasonally adapted.

Possible reasons for the seasonal patterns observed may relate to nutritional variability. During the long rainy season (March to June), physical activity diminishes due to frequent rainfall, and people tend to consume high-fat and high-calorie food while awaiting the harvest. Therefore, it is also possible that increased caloric intake could increase hyperglycaemia and the emergence of symptoms. West showed in 1978 that the decrease in the daily intake of calories in World War II Europe was associated with a decrease in the incidence of diabetes [37]. Also, viral infections have been implicated for many years as a possible environmental factor in the aetiology of type 1 and type 2 diabetes [27, 38]. There appears to be a seasonal variation in the onset of acute type 1 diabetes, with a peak in the autumn [7, 39]; diseases with seasonal incidences are often caused by viral infections. There also have been numerous anecdotal reports of a temporal association between viral infections and the development of diabetes in some patients [40]. Little information are available on the seasonality of viral infections in the tropics, although the increase in hospital admission rates for respiratory diseases, including asthma, makes it likely that there is an increase in viral-related respiratory infections during the rainy season. The development of diabetes has also been associated with viral hepatitis in West Africa, but the stress due to the infection may be a confounding factor in the exacerbation of pre-existing hyperglycaemia [41].

It is possible that in the rainy season which is also associated with infectious diseases, seasonal patterns observed in our study may also relate to diseases such as malaria. Diabetes is frequently associated with infections as seen in clinical practice. Of them, bacterial, viral and fungal infections are common [42]. Though bacterial infection remains the most common precipitating factor of diabetes keto-acidosis worldwide, malaria has been emphasized as a precipitant in developing countries [43]. Ketosis, precipitated by falciparum malaria has already been reported [44]. In African populations, there is general agreement on the role of environemental factors such as diet, viral infections in diabetes, but malaria has not been proved to be a causal factor. Therefore, in tropical countries, malaria should be looked for in patients with diabetes. In diabetic animal models, malaria infection dramatically lowers blood glucose [45]. Unexplained hypoglycemia caused by falciparum malaria in patients with diabetes before initiation of quinine therapy has been reported [46, 47]. Since little information is available on the profile of falciparum malaria infection in diabete patients in tropical areas. A possible role of malaria in the sesonality of diabetes cannot be excluded.

The major limitations of the study are that the types of diabetes and age at diagnosis or onset were not reported. Screening for different types of diabetes may have strengthened the findings. The study was also limited by the time period of the data collection (9 years). Over such a time period, changes in unidentified environmental factors could have confounded the results. Another limitation of the present study is that the narrow sample which is not representative of the total population in Cameroon and therefore cannot be generalized to that population. Moreover, data collection did not include the geographical origin of each participant, a factor that could also have influenced the results. A previous study in Cameroon, showed that the prevalence of diabetes is higher in urban than in rural areas [48]. Further studies, particularly cohort studies with larger numbers of diabetes patients, are required to draw definite conclusions regarding the seasonality of diabetes hospitalization admission rate, thus possibly offering data on the etiology and epidemiology of this disease. A comparison with viral epidemiology data during the same period could likely yield useful results.

## Conclusion

In conclusion, the variation in the prevalence of diabetes is almost superimposable to that of precipitation; the hospital admission rate of diabetes patients is slightly higher during the rainy season. Seasonality of diabetes hospital admissions rates seens to encountered in the Cameroonian population. This study is the first to explore the seasonality of diabetes in Cameroon. Nevertheless, it should be stated that this is an observational study on the seasonality of acute diabetic flares, from which no conclusions can be drawn regarding the pathophysiology of diabetes.

## References

[1] International Diabetes Federation (IDF). Diabetes Atlas 6th edition. Brussels: International Diabetes Federation; 2013 [October 6, 2014]; Available from: http://www.idf.org/sites/default/files/EN_6E_Atlas_Full_0.pdf. Accessed 24 May 2016.

[2] Danaei G, Finucane MM, Lu Y, Singh GM, Cowan MJ (2011). National, regional, and global trends in fasting plasma glucose and diabetes prevalence since 1980: systematic analysis of health examination surveys and epidemiological studies with 370 country-years and 2.7 million participants. Lancet.

[3] L’Équipe de rédaction principale GIEC , Pachauri RK, Andy Reisinger A. Bilan 2007 des changements climatiques : Rapport de synthèse. http://www.ipcc.ch/pdf/assessment-report/ar4/syr/ar4_syr_fr.pdf. Accessed 24 May 2016.

[4] Gherard R (1988). Paysages et milieux épidémiologiques dans l’espace ivoiro-burkinabé.

[5] No authors listed (1998). UK prospective diabetes study. IV, Characteristics of newly presenting Type 2 diabetic patients: Male preponderance and obesity at different ages. Diabet Med.

[6] Papoz L, Williams R, Fuller J, eds. Le diabète en Europe Paris; INSERM. John LIBBEY, 1994;15–31.

[7] Adams F (1926). The seasonal variation in the onset of acute diabetes. The age and sex factors in 1000 diabetic patients. Arch Intern Med.

[8] Gray RS, Duncan LJ, Clarke BF (1979). Seasonal onset of insulin dependent diabetes in relation to sex and age at onset. Diabetologia.

[9] Karayanni C, Anastasiadou V, Spyropoulou M (1993). Genetic predisposition and IDDM in Greece. Genet Couns.

[10] Bartsocas CS, Dacou-Voutetakis C, Damianaki D (1998). Epidemiology of childhood IDDM in Athens: trends in incidence for the years 1989-1995.Eurodiab AcE G1 Group. Diabetologia.

[11] Levy-Marchal C, Patterson C, Green A (1995). Variation by age group and seasonality at diagnosis of childhood IDDM in Europe. The EURODIAB AcE Study Group. Diabetologia.

[12] McKinney PA (2011). Seasonality of birth in patients with childhood Type I diabetes in 19 European regions. Diabetologia.

[13] Patterson CC, Smith PG, Webb J, Heasman MA, Mann JI (1988). Geographical variation in the incidence of diabetes mellitus in Scottish children during the period 1977-1983. Diabet Med.

[14] Green A, Patterson CC (2001). Trends in the incidence of childhood-onset diabetes in Europe 1989-1998. Diabetologia.

[15] Pocecco M, Nassimbeni G (1993). Distribution of new cases of insulin-dependent diabetes mellitus (IDDM) by age, sex, seasonality, and clinical characteristics at onset in youngsters from the Friuli Venezia Giulia region from 1987 to 1990. Pediatr Med Chir.

[16] Scott RS, Brown LJ, Darlow BA, Forbes LV, Moore MP (1992). Temporal variation in incidence of IDDM in canterbury, New Zealand. Diabetes Care.

[17] Soltész G, Madácsy L, Békefi D, Dankó I (1989). Incidence of childhood diabetes in Hungary. Orv Hetil.

[18] Waldhoer T, Schober E, Tuomilehto J (1997). Long-term patterns in seasonality of insulin-dependent diabetes mellitus diagnosis in Austrian children. J Clin Epidemiol.

[19] Kida K, Mimura G, Ito T, Murakami K, Ashkenazi I, Laron Z (2000). Incidence of Type 1 diabetes mellitus in children aged 0-14 in Japan, 1986-1990, including an analysis for seasonality of onset and month of birth: JDS study. The Data committee for childhood Diabetes of the Japan Diabetes Society (JDS). Diabet Med.

[20] Maria IK, Vazeou A, Delis D, Bozas E, Thymelli I (2011). Seasonal variation of type 1 diabetes mellitus diagnosis in Greek children. Hormones (Athens).

[21] Imagawa A, Hanafusa T, Makino H, Miyagawa JI, Juto P (2005). High titres of IgA antibodies to enterovirus in fulminant type-1 diabetes. Diabetologia.

[22] Bartsocas CS, Lab M, Spyrou N, Krikelis N, Serié C (1982). Are viral studies indicated in juvenile-onset diabetes?. Padiatr Padol.

[23] Bartsocas CS, Papadatos CJ, Lab M, Spyrou N, Krikelis B (1982). Coxsackie B viruses and autoimmune diabetes. J Pediatr.

[24] Cantorna MT (2000). Vitamin D and autoimmunity: is vitamin D status an environmental factor affecting autoimmune disease prevalence?. Proc Soc Exp Biol Med.

[25] Lecube A, Hernández C, Genescà J, Simó R (2006). Glucose abnormalities in patients with hepatitis C virus infection: epidemiology and pathogenesis. Diabetes Care.

[26] Sun Y, Pei W, Wu Y, Yang Y (2005). An association of herpes simplex virus type 1 infection with type 2 diabetes. Diabetes Care.

[27] Sobngwi E, Choukem SP, Agbalika F, Blondeau B, Fetita LS (2008). Ketosis-Prone Type 2 Diabetes Mellitus and Human Herpesvirus 8 Infection in Sub-Saharan Africans. JAMA.

[28] Moltchanova EV, Schreier N, Lammi N, Karvonen M (2009). Seasonal variation of diagnosis of Type 1 diabetes mellitus in children worldwide. Diabet Med.

[29] MacDonald MJ, Liston L, Carlson I (1987). Seasonality in glycosylated haemoglobin in normal subjects. Does seasonal incidence in insulin-dependent diabetes suggest specific aetiology?. Diabetes Care.

[30] Fleegler FM, Kromann H, Christy M, Andersen OO, Nerup J (1979). Age, sex and season of onset of juvenile diabetes in different geographical areas. Pediatrics.

[31] Omar MA, Asrnal AC (1984). Patterns of diabetes mellitus in young Africans and Indians in Natal. Trop Ceogr Med.

[32] McLarty DG, Yusafali A, Swai AB (1989). Seasonal incidence of diabetes mellitus in tropical Africa. Diabet Med.

[33] American Diabetes Association (2011). Standards of medical care in diabetes—2011. Diabetes Care.

[34] Assongmo T. Les quartiers marginaux de l’agglomération de Yaoundé. Logique de construction et problèmes d’aménagement. Toulouse: Thèse de Doctorat de Géographie N.R.

[35] Ahmed AM (2006). Les soins du diabète au Soudan : Problèmes émergents et besoins aigus. Diabetes Voice.

[36] Ahmed AM, Ahmed NH, Abdalla ME (2000). Pattern of hospital mortality among diabetic patients in Sudan. Pract Diabetes Int.

[37] West KM (1978). Epidemiology of Diabetes and Its Vascular Lesions.

[38] Lecube A, Hernández C, Genescà J, Simó R (2006). Proinflammatory cytokines, insulin resistance, and insulin secretion in chronic Hepatitis C patients. Diabetes Care.

[39] Gamble DR, Taylor KW (1969). Seasonal incidence of diabetes mellitus. BMJ.

[40] Yoon JW, Jun HS, Pickup J, Williams G (2002). Viruses in the pathogenesis of type 1 diabetes. Textbook of diabetes, 3rd ed.

[41] Adi FC (1974). Diabetes mellitus associated with epidemic of infectious hepatitis in Nigeria. Br Med J.

[42] Pozzili P, Siognore A, Leslie RD, Alberti KGMM, Zimmet P, Defronzo R, Keen H (1997). Infections, Immunity and Diabetes. International Text Book of Medicine.

[43] Rwija HJ, Swai ABM, McLarty D (1986). Failure to diagnose diabetic ketoacidosis in Tanzania. Diabet Med.

[44] Maji D, Mukherjee S (1995). Diabetic ketoacidosis and infection. J Diab Assoc Ind.

[45] Elased K, DeSouza JB, Playfair JH (1995). Blood stage malaria in diabetic mice. Clin Exp Immunol.

[46] Shalev O, Tsur A, Rahav G (1992). Falciparum malaria induced hypoglycemia in a diabetic patient. Postgrad Med J.

[47] Jikki PN, Krishnamurty CM (1997). Hypoglycemia in a patient with diabetes mellitus with falciparum malaria. J Assoc Phys Ind.

[48] Sobngwi E, Mbanya JC, Unwin NC, Kengne AP, Fezeu L (2002). Physical activity and it relationship with obesity. Hypertension and diabetes in urban and rural Cameroon. Int J Obes Relat Metab Disord.

